# Metazoan parasites of California sea lions (*Zalophus californianus*): A new data and review

**DOI:** 10.1016/j.ijppaw.2018.09.001

**Published:** 2018-09-05

**Authors:** Tetiana A. Kuzmina, Terry R. Spraker, Olena Kudlai, Olga I. Lisitsyna, Svitlana O. Zabludovskaja, Grzegosz Karbowiak, Christine Fontaine, Roman Kuchta

**Affiliations:** a I. I. Schmalhausen Institute of Zoology NAS of Ukraine, 15, Bogdan Khmelnitsky Street, Kyiv, 01030, Ukraine; b Colorado State University, Diagnostic Laboratory, Department of Microbiology, Immunology and Pathology, College of Veterinary Medicine and Biomedical Sciences, Colorado State University, Fort Collins, CO, 80526, USA; c Institute of Ecology, Nature Research Centre, Akademijos, 2, 08412, Vilnius, Lithuania; d Water Research Group, Unit for Environmental Sciences and Management, Potchefstroom Campus, North-West University, Potchefstroom, 2520, South Africa; e Witold Stefański Institute of Parasitology of Polish Academy of Sciences; Twarda Street, 51/55, 00-818, Warsaw, Poland; f The Marine Mammal Center, 2000 Bunker Road, Fort Cronkhite, Sausalito, CA, 94965-2619, USA; g Institute of Parasitology, Biology Centre of the Czech Academy of Sciences, Branišovská 31, 37005, České Budějovice, Czech Republic

**Keywords:** Marine mammals, Nematoda, Cestoda, Trematoda, Acanthocephala, Biodiversity

## Abstract

The population of California sea lion *Zalophus californianus* (CSL) has steadily increased during the last several decades. Despite extensive research addressing CSL biology and ecology performed during the last decades, there has been a minimal number of published papers documenting their parasite fauna. Our objective was to analyze the actual list of the metazoan parasites reported from CSLs and add new data on the age-related differences in the prevalence and biodiversity of the parasite community. There have been 33 species recorded but this study considers only 24 of them valid. Among them, 11 species are specific parasites of CSLs and 13 species are not specific. Additional species represent accidental infections or misidentifications. In total, 6653 helminths and 847 mites were collected and identified from 34 CSLs for this study. Six species of nematodes, *Anisakis simplex sensu lato s*. *l*. (prevalence 41%; intensity 7.6), *Contracaecum ogmorhini s*. *l*. (38%; 269.6), *Pseudoterranova decipiens s*. *l*. (29%; 33), *P. azarazi* (9%; 2.7), *Acanthocheilonema odendhali* (15%; 3.5) and *Parafilaroides decorus* were found. Two species of cestodes, *Diphyllobothrium* sp. (38%; 8.5) and *Anophryocephalus* sp. (15%; 14.6) represent novel undescribed species. Two species of trematodes, *Apophallus zalophi* (18%; 19.7) and *Zalophotrema hepaticum* (12%; 39.2), and five species of acanthocephalans, *Corynosoma obtuscens* (68%; 100.8), *C. strumosum* (53%; 4.6), *Andracantha phalacrocoracis* (3%; 1), *Andracantha* sp. (9%; 4.3) and *Profilicollis altmani* (6%; 8.5) were found. Mites *Orthohalarchne attenuata* (prevalence 85%) were found in the nasal cavity, while *O. diminuata* (21%) parasitized in the trachea and bronchi. The highest levels of infection with nematodes and trematodes were found in adult CSLs (3–16 years old), whereas the highest level of infection with acanthocephalans was found in young CSLs (pups and yearlings).

## Highlights

• 24 species of metazoan parasites confirmed.
• 17 species documented.
• Significant differences in species diversity observed in pups and adult CSLs.

## Graphical abstract

**Figure:**
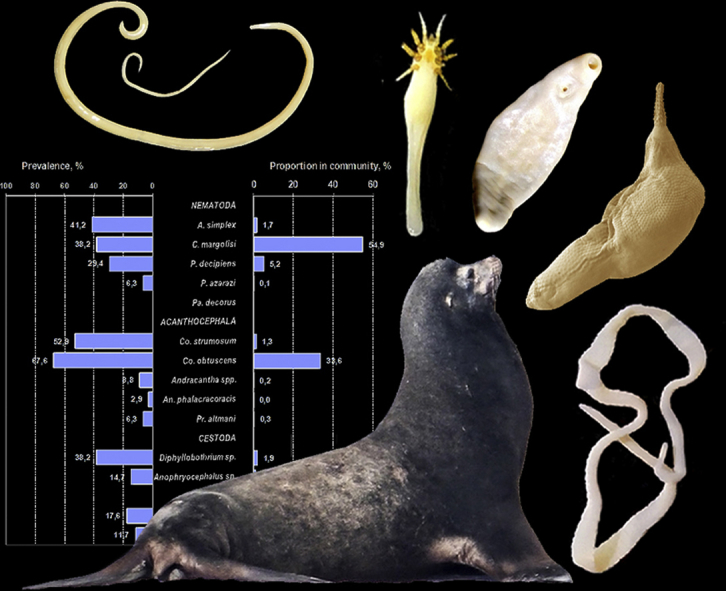

## 1 Introduction

The parasite fauna of marine mammals is relatively well known, but most of the studies were performed around 50 years ago (Delyamure, 1955; Dailey and Brownell, 1972; etc.); and more recent detailed studies are mostly missing. There are several checklists that represent parasites of marine mammals which include California sea lions *Zalophus californianus* (CSL) (Delyamure, 1955; King, 1964; Dailey and Brownell, 1972; Margolis and Dailey, 1972; Dailey, 1975; Felix, 2013), but most reports include records based on incorrect parasite identification or accidental infections. Moreover, these incorrect data have been repeated in the literature without any corrections or additions of new data since the study of Delyamure (1955). Clarification of these records requires examination of new well fixed material or re-examination of the vouchers deposited in collections, but they are mostly missing. Such studies existed on the parasite fauna of northern fur seals *Callorhinus ursinus* at St. Paul Island, Alaska, where more than 27,000 specimens of parasites from 756 subadult male seals were found, described and vouchers deposited in the collections (Kuzmina et al., 2012, 2014, 2015, 2018; Hernández-Orts et al., 2015, 2018).

The present study is attempting to verify the contemporary data on the helminth fauna of a well-known pinniped species, *Z. californianus*, whose population has grown in recent decades. Since the Marine Mammal Protection Act was signed in 1972, the population of CSLs has increased from approximately 50,000 to 340,000 individuals (Carretta et al., 2007; McClatchie et al., 2016). Previously, only one species, *Z. californianus*, with three subspecies was recognized within the genus *Zalophus* Gill, 1866, but new studies considered these subspecies to be valid species (Wolf et al., 2007). Therefore, the reported records of the parasites from Galápagos sea lions (*Zalophus wollebaeki*) and Japanese sea lion (*Zalophus japonicus*) are not included in this study (Nagasawa, 1999; Dailey et al., 2005). Despite intensive studies addressing the biology and ecology of CSLs performed over the last four decades (Laake et al., 2016), only a few studies on the CSL parasite fauna have been published (Dailey and Hill, 1970; Dailey, 1978; Stroud and Dailey, 1978).

The first described parasite of CSLs, the liver fluke *Zalophotrema hepaticum* Stunkard et Alvey, 1929, was collected from a CSL that died in the Aquarium of the New York Zoological Society (Stunkard and Alvey, 1929, 1930). Based on available published data, currently more than 30 species of metazoan parasites have been reported in CSLs (Delyamure, 1955; King, 1964; Dailey and Brownell, 1972; Dailey, 1975; Mattiucci et al., 2003; Felix, 2013; Kuzmina and Kuzmin, 2015; Mariaux et al., 2017; Waeschenbach et al., 2017) (see Table 1). However, most of these published records are based on unclear identification of the parasites without their documentation and depositing of the voucher specimens in collections. Moreover, at least five species of parasitic protists (*Toxoplasma gondii* (Nicolle et Manceaux, 1908), *Sarcocystis neurona* Dubei, Davis, Speer et al., 1991, *Neospora* sp., *Cryptosporidium* sp. and *Giardia* sp.) have also been reported in CSLs (Carlson-Bremer et al., 2012, 2015; Adell et al., 2014; Girard et al., 2015).

**Table 1:** List of parasites found in California sea lions (*Zalophus californianus*) according to published records combined with the results of the present study. Species found in this study are in bold.

Species	Type host/Site^a^	References	Specificity^b^
TREMATODA^e^			
*Apophallus zalophi* (as *Pricetrema*)	CSL/I	Price, 1932,^d^; Ciurea, 1933; Dailey and Hill, 1970; Stroud and Dailey 1978	SP
*Galactosomum ubelakeri* (as *Stictodora*)	CSL/I	Dailey, 1969; Dailey and Hill 1970	SP
***Zalophotrema hepaticum***	CSL/I	Stunkard and Alvey, 1930; Price, 1932; Ezzat et al., 1958, d; Sweeney and Gilmartin, 1974; Sweeney, 1973; Stroud and Dailey 1978; Schroeder and Wegeforth, 1935; Fleischman and Squires, 1970	SP
CESTODA^f^			
***Anophryocephalus* sp.**^c^	CSL/I	Mariaux et al., 2017	SP
***Diphyllobothrium* sp.**^c^	CSL/I	Stunkard, 1965; Waeschenbach et al., 2017	SP
NEMATODA^g^			
***Acanthocheilonema odendhali*** (as *Dipetalonema*)	CSL/T	Perry, 1967; Perry and Forrester, 1971; Dailey and Hill, 1970; Dailey, 1975; Stroud and Dailey, 1978	SP
***Anisakis simplex****s.l.* (syn. *A. similis*)	*Phocena phocena*/S	Schroeder and Wegeforth, 1935; Yamaguti, 1961; Stroud and Dailey, 1978	NS
*Contracaecum osculatum*	*Phoca vitulina***/**S	Herman, 1942, d; Margolis, 1954; Flores-Barroeta et al., 1961; Dailey and Hill, 1970; Fleischman and Squires, 1970; Sweeney and Gilmartin, 1974; Ridgway et al., 1975; Dailey, 1978; Stroud and Dailey, 1978	NS
***Contracaecum ogmorhini****s.l.*	*Hydrurga leptonyx*/S	Fagerholm and Gibson, 1987; Dailey, 1978; D'Amelio et al., 1994; Zhu et al., 2001	NS
*Contracaecum margolisi*	CSL/S	Mattiucci et al., 2003	NS
*Otostrongylus circumlitus*	*P. vitulina*/P	Stroud and Dailey, 1978; Kelly et al., 2005	NS
***Parafilaroides decorus***	CSL/P	Dougherty and Herman, 1947; Johnson and Ridgway, 1969; Fleischman and Squires, 1970; Dailey, 1970; Dailey and Hill, 1970; Sweeney and Gilmartin, 1974; Schroeder et al., 1973; Ashizawa et al., 1978; Geraci, 1978; Dailey, 1978; Stroud and Dailey, 1978	SP
***Pseudoterranova decipiens****s*. *l*.	*Pagophilus groenlandicus*/S	Herman, 1942, d; Stroud and Dailey, 1978; Paggi et al., 1991	NS
***Pseudoterranova azarazi***	*Phoca fasciata*/S	new host record	NS
*Uncinaria lyonsi* (as *Uncinaria* sp.)	CSL/I	Dailey and Hill, 1970; Dailey, 1978; Lyons et al., 1997, 2000, 2003, 2016; Kuzmina and Kuzmin, 2015	SP
ACANTHOCEPHALA			
***Andracantha phalacrocoracis***	*Phalacrocoracis pelagicus*/I	new host record	NS
***Andracantha* sp.**^c^	CSL/I		NS
***Corynosoma obtuscens***	CSL/I	Lincicome, 1943, d; Van Cleave, 1953, d; Dailey and Hill, 1970; Dailey, 1978	SP
***Corynosoma strumosum*** (syn. *C. osmeri*)	*P. vitulina*/I	Van Cleave, 1953	NS
***Profilicollis altmani***	*Melanitta perspicillata*/I	new host record	NS
ARTHROPODA			
*Antarctophthirus microchir*	*Phocarctos hookeri*/K	Ferris, 1916, 1934, 1951; Jellison, 1952; Dailey and Hill 1970; Dailey, 1978	NS
*Demodex zalophi*	CSL/K	Dailey and Nutting, 1980 (as *Demodex* sp.)	SP
***Orthoharachne diminuata*** (syn. *O. letalis*)	CSL/P	Doetschman, 1944; Newell, 1947; Margolis, 1956; Dahme and Popp, 1963; Dailey and Hill, 1970; Furman, 1977	SP
***Orthohalarchne attenuata*** (syn. *O. zalophi*)	*Callorhinus ursinus*/N	Dailey and Hill, 1970; Dailey, 1978; Stroud and Dailey, 1978	NS

References from checklists of Delyamure (1955), King (1964), Dailey and Brownell (1972), Margolis and Dailey (1972), Dailey (1975) and Felix (2013) are were not included into the list.

a Type host/Site of infection in CSL: I – intestine; K – skin; L – liver; N – nasal cavity; P – pulmonary (lung) or trachea and bronchi; S – stomach; T – subcutaneous tissues.

b Host specificity to CSL: NS – not specific; SP – specific.

c New undescribed species.

d Record from CSL captured in zoo.

e Additional species were considered as accidental parasites: *Heterophyes heterophyes*, *Schistosoma haematobium*, *Schistosoma mansoni* known from humans by Ezzat et al. (1958), ^d^ and *Nanophyetus salmincola* and *Stephanoprora denticulata* (as *Mesorchis*) known from dogs by Stroud and Dailey (1978) and Price, 1932^d^ respectively.

f *Adenocephalus pacificus* (as *Diphyllobothrium*) was misidentified with new species of *Diphyllobothrium* by several authors (Margolis, 1956; Dailey and Hill, 1970; Sweeney, 1973; Dailey, 1978).

g *Acanthocheilonema spirocauda* (as *Skrjabinia*) and *Dujardinia* sp. known from other pinnipeds were misidentified by Taylor et al., 1961) and Herman, 1942^d^ respectively; *Dirofilaria immitis* (syn. *D. fausti*) known from dogs was considered as accidental parasite reported by Faust (1937),^d^; White, 1975,^d^; Sato et al., 2002,^d^ and Alho et al., 2017^d^.

The first survey on metazoan parasites of CSLs was published by Dailey and Hill (1970) and was based on 14 animals collected from southern to central California. A few years later, the survey was updated (Dailey, 1978; Stroud and Dailey, 1978) adding only one new host record for the trematode *Nanophyetus salmincola* (Chapin, 1926). They found nine species of helminths and three species of parasitic arthropods (Table 1). Later, the data were repeated in several checklists and review reports on parasites of marine mammals (Dailey and Brownell, 1972; Margolis and Dailey, 1972; Dailey, 1975; Felix, 2013). Recently, a hookworm recorded from CSLs was extensively studied (Lyons et al., 1997, 2000, 2011, 2016; Spraker et al., 2004) and considered as a new species *Uncinaria lyonsi* (Kuzmina and Kuzmin, 2015). Moreover, CSLs were recently reported to be a new host for *Otostrongylus circumlitus* (Railliet, 1899) (Kelly et al., 2005). However, studies on prevalence and species diversity of metazoan parasites in CSLs have not been performed during the last four decades. The objective of the present study is to revise the published data on the parasites of CSLs and update the list of metazoan parasites with the inclusion of new data. Examination of any possible age-related differences in the prevalence and biodiversity of the parasites in CSLs was also performed.

## 2 Material and methods

Our studies were carried out in February–March of 2015, 2016 and 2018 at The Marine Mammal Center (TMMC), Sausalito, California, USA. Thirty-four CSLs from 8–9 months to 16 years of age, which were found stranded on the Pacific coast of central California and died from different causes at TMMC were necropsied using a standard procedure (Spraker, 1985). All sea lions examined were separated into three age groups: pups 8–9 month old (n = 10) and 10–11 months old (n = 12), yearlings (1.8 years old) (n = 4) and adults (3–16 years old) (n = 8). The ages of these animals were approximated by their over-all body size, size of their teeth and time they should have been born.

Intestinal contents were collected from different parts of the CSLs intestines and washed through a sieve with nominal openings of 0.297 mm to avoid losing small helminths. Gastrointestinal parasites (n = 6,653) and nasal mites (n = 847) were collected manually, washed in saline or tap water, fixed in 70% non-denatured ethanol and identified under a light microscope Zeiss Axio Imager M1 based on their morphology. Prior to identification all nematodes were clarified in lacto-phenol solution (25% phenol, 25% lactic acid, 25% glycerine, 25% distil water) for more than 3 h. Trematode and cestode specimens were stained with acetocarmine or with Schuberg’s hydrochloric carmine, dehydrated in a graded ethanol series, cleared in clove oil, and mounted permanently in Canada balsam. All acanthocephalans were mounted in Berlese's medium.

Data summaries and descriptive analyses were calculated using Microsoft™ Excel and Paleontological Statistics Software (PAST) (Hammer et al., 2001). The Mann-Whitney test was used to analyze differences in helminth infections of the CSL of different age groups. Proportion of the helminth species in the parasite community was calculated as the relation of the number of specimens of the certain species to the total number of helminths collected in percentage (%).

## 3 Results

Based on detailed analysis of all previously published as well as new data, 24 species were documented to be valid metazoan parasites of CSL (Table 1). From them, 11 species were specific parasites of CSLs, 13 species were not specific and able to infect other hosts. The rest of the 9 previously reported species were considered as accidental infections (such as trematodes distributed in Africa and Asia), or misidentifications (Table 1). Seventeen species of metazoan parasites were collected from CSLs in the present study (Table 2). The most common were nasal mites *Orthohalarchne attenuata* (Banks, 1910) found in 85% of dissected hosts. This species was followed by *Corynosoma obtuscens* Lincicome, 1943 (67%), *C. strumosum* (Rudolphi, 1802) (53%) and *Anisakis simplex s*. *l*. (Rudolphi, 1809) (41%) (Table 2). However, the intensity of infections was the highest with the nematode *Contracaecum ogmorhini* Johnston et Mawson, 1941 (up to 1,530 specimens per host; mean intensity 269.6), followed by acanthocephalan *C. obtuscens* (1.200; 100.8) and the nematode *Pseudoterranova decipiens s*. *l*. (Krabbe, 1868) (260; 33.0) (Table 2).

**Table 2:** Parasites found in California sea lions (*Zalophus californianus*) in the Marine Mammal Center (TMMC), Sausalito, California.

Parasites	Organ infected^b^	No.^c^	Prevalence %	Intensity			
				Min	Max	Average	Median
**Nematoda**							
*Anisakis simplexs. l.*	S; Is	13	41	1	41	7.6	4.5
*Contracaecum ogmorhini*	S; Is	13	38	1	1530	269.6	118
*Pseudoterranova decipienss. l.*	S; Is	10	29	1	260	33.0	6
*Pseudoterranova azarazi*	S; Is	3	9	1	6	2.7	1.5
*Acanthocheilonema odendhali*	K	5	15	1	5	3.5	4
*Parafilaroides decorus*	P	2^a^	ND^a^	ND			
**Trematoda**							
*Apophallus zalophi*	Is	6	18	1	56	19.7	6.5
*Zalophotrema hepaticum*	L	4	12	5	91	39.2	20
**Cestoda**							
*Diphyllobothrium* sp.	Is	13	38	1	30	9.5	6
*Anophryocephalus* sp.	Is	5	14	1	35	14.6	8
**Acanthocephala**							
*Corynosoma strumosum*	Il; Is	17	53	1	24	4.6	2
*Corynosoma obtuscens*	Il; Is	21	68	1	1200	100.8	17
*Andracantha phalacrocoracis*	Il; Is	1	3	1	1	1	1
*Andracantha* sp.	Il; Is	3	9	1	9	4.3	3
*Profilicollis altmani*	Is	2	6	3	14	8.5	8.5
**Acarina**							
*Orthohalarchne diminuata*	T	7	21	ND			
*Orthohalarchne attenuata*	N	29	85	ND			

a Lungs, nasal cavities and skin were not examined in the most of CSLs; ND – not detected.

b Organ infected: Il - large intestine; Is - small intestine; K - under skin; L - liver; N - nasal cavity and pharinx; P - pulmo (lung); S - stomach; T - trachea and bronchi.

c Number of CSL infected.

**Fig. 1:**
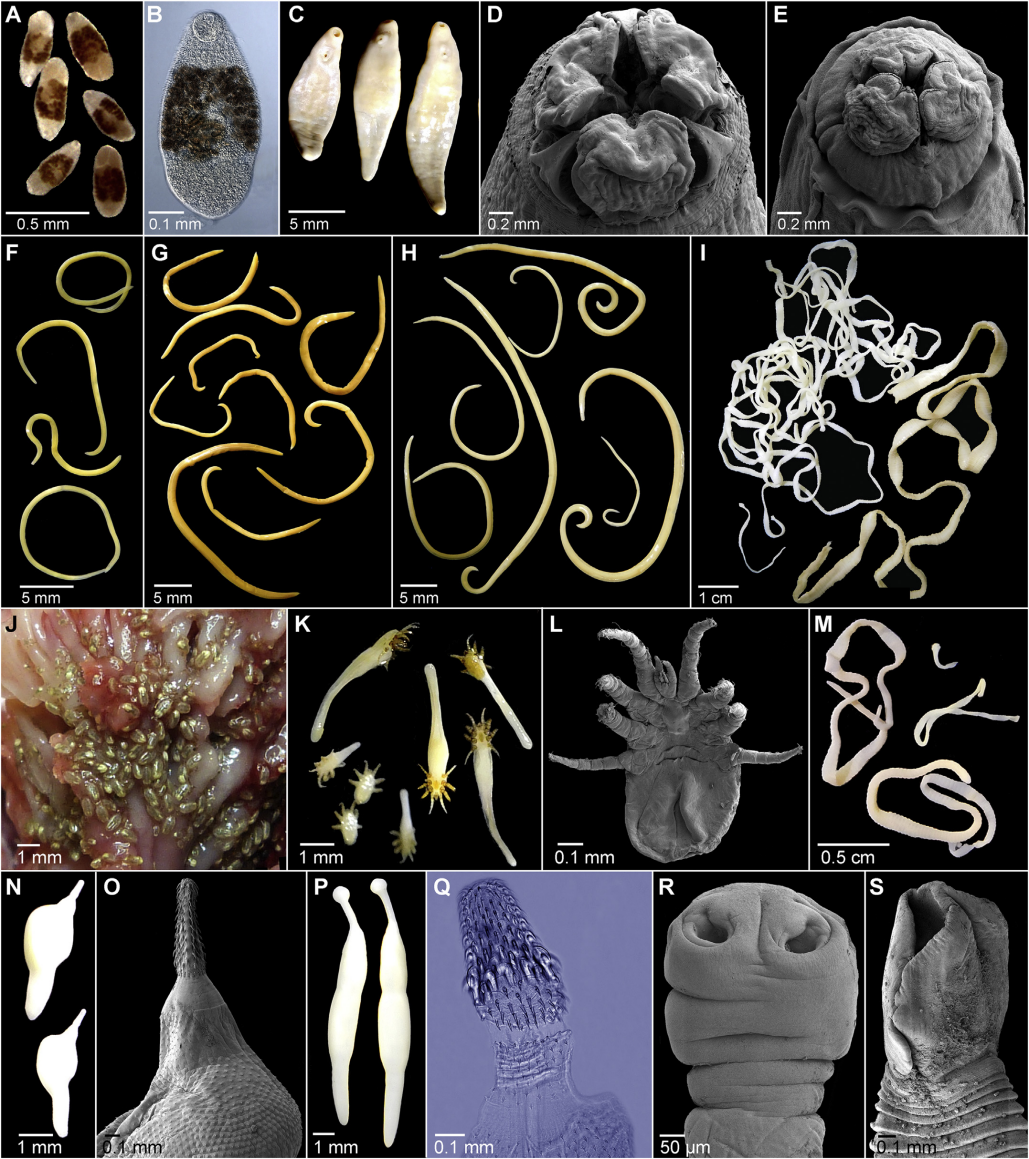
Microphotographs of the metazoan parasites of California sea lions *Zalophus califronianus*. A, B – *Apophallus zalophi*, intestine (Digenea), C – *Zalophotrema hepaticum*, liver (Digenea), D, G – *Contracaecum ogmorhini s. l.*, stomach (Nematoda), E, H – *Pseudoterranova decipiens s*. *l*., stomach (Nematoda), F – *Anisakis simplex s*. *l*., stomach (Nematoda), I, S – *Diphyllobothrium* sp., intestine (Cestoda), J, K – *Orthohalarchne attenuata*, nasal cavity (Acarina), L – *Orthohalarchne diminuata* (Acarina), M, R – *Anophryocephalus* sp., intestine (Cestoda), N, O – *Corynosoma obtuscens*, intestine (Acanthocephala), P, Q – *Profilicollis altmani*, intestine (Acanthocephala). A, C, F–I, K, M, N, P – under dissecting scope. B, Q – at light microscope. D, E, O, R, S, – at scanning electron microscope. J – *in situ*.

Nematodes were represented by six species of the families Anisakidae (*A. simplex s*. *l*. (Rudolphi, 1809), *C. ogmorhini s*. *l*. Johnston et Mawson, 1941, *P. decipiens s*. *l*. and *P. azarazi* (Yamaguti et Arima, 1942)), Filariidae (*Acanthocheilonema odendhali* Perry, 1967), and Filaroididae (*Parafilaroides decorus* Dougherty et Herman, 1942) (Fig. 1).

Five species of acanthocephalans (Polymorphidae) were found, including two species with new host records for immature *Andracantha phalacrocoracis* (Yamaguti, 1961) and *Profilicollis altmani* (Perry, 1967). In addition, we found only immature specimens related to the genus *Andracantha* Schmidt, 1975, but morphologically distinct from all known species of this genus which indicates the presence of a new undescribed species (Table 2; Fig. 1).

Only two species of trematodes, namely *Apophallus zalophi* Price, 1932 (Heterophyidae) in the intestine and *Zalophotrema hepaticum* Stunkard et Alvey, 1929 (Brachycladiidae) in the liver, were found with relatively low prevalence and intensity (Table 2; Fig. 1).

Two previously undescribed cestode species that were briefly reported by one of the present author were recognized in this study. One belonging to the genus *Diphyllobothrium* Cobbold, 1858 (Diphyllobothriidea) which was previously misidentified as *D. pacificum* (Nybelin, 1931), and the second belonging to the genus *Anophryocephalus* Baylis, 1922 (Tetrabothriidea) (Table 1; Hérnandez-Orts et al., 2015; Waeschenbach et al., 2017; Mariaux et al., 2017). Thirteen CSLs (3 pups, 3 yearlings and 7 adults) were infected with *Diphyllobothrium* sp. and five (3–16 years old) were infected by *Anophryocephalus* sp. (Table 2).

*Diphyllobothrium* sp. 1 of Waeschenbach et al. (2017) is conspecific with our present material. They are large worms up to 2.3 m long and 0.8 cm wide with typical lanceolated scolex with two bothria and typical strobilar morphology of the genus *Diphyllobothrium* (Fig. 1). The specimens are not conspecific with any other described species of the genus and represent a new undescribed species that was indicated also by molecular data (Delyamure et al., 1985; Waeschenbach et al., 2017; Kuchta and Scholz, 2017).

*Anophryocephalus* sp. of Mariaux et al. (2017) are small tapeworms up to 7 cm long and 1 mm wide with a typical spherical scolex with 4 bothridia and typical strobilar morphology of the genus *Anophryocephalus* (Fig. 1). This is the first record of the genus *Anophryocephalus* in CSLs and outside of the Arctic and Subarctic regions (Mariaux et al., 2017). The description of both cestodes will be published in a separate paper.

Ectoparasites were represented by two species of mites (Halarachnidae), namely *Orthohalarchne attenuata* and *O. diminuata* Doetschman, 1944, which were observed in the nasal cavity and/or trachea and bronchi of all age groups of CSLs (Table 2). The intensity of infection by these mites was not determined; although only single mites were observed in pups, while hundreds of mites were found in the nasal cavity of adult sea lions. Most of the mites of both species collected in the CSLs were adults; only a few nymphs were observed.

Acanthocephalans were the dominant group of helminths found in this study with a prevalence of 74%; they accounted for up to 36% of the total helminths found. The most common species was *C. obtuscens* which is a specific parasite of CSLs (Table 2).

**Fig. 2:**
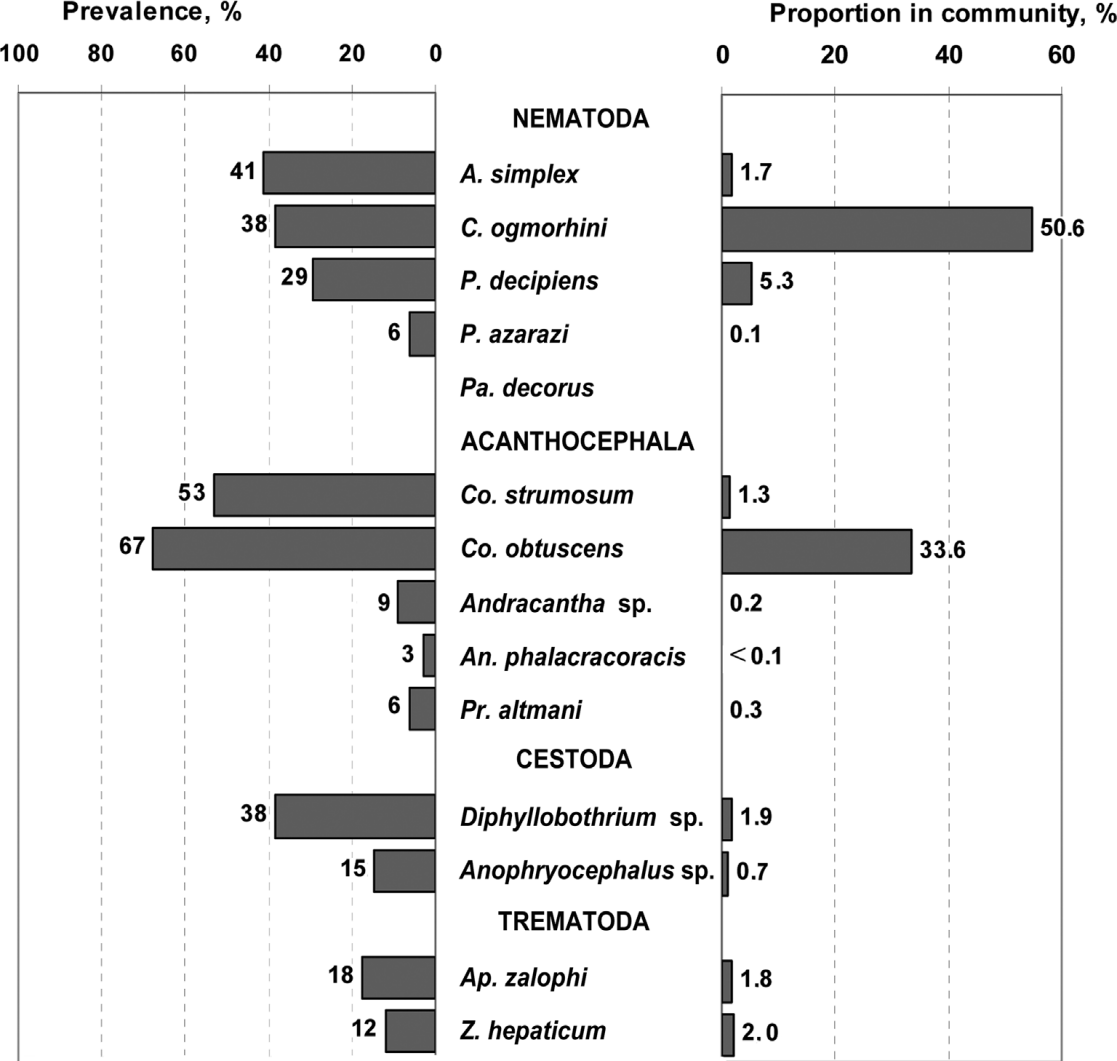
Prevalence and proportion of separate species in the gastrointestinal helminth community of California sea lions (*Zalophus californianus*). Abbreviations of the genera: *A* – *Anisakis*, *An* – *Andracantha*, *Ap* – *Apophallus*, *C* – *Contracaecum*, *Co* – *Corynosoma*, *P* – *Pseudoterranova*, *Pa* – *Parafilaroides*, *Pr* – *Profilicollis*, *Z* – *Zalophotrema*.

The prevalence of nematodes was lower (59%) than that of acanthocephalans; however, they amounted to 62% of the total helminth number with *C. ogmorhini s*. *l*. as the dominant nematode species (Fig. 2). Fifty-one percent of *A. simplex s*. *l*. specimens were larvae; while for species of the genus *Pseudoterranova* Mozgovoi, 1951 and for *C. ogmorhini s*. *l*. larval stages were less numerous (26% and 25% of specimens, respectively; data not shown).

Prevalence of cestodes and trematodes were 41% and 27% respectively, whereas their proportion in the helminth community was only 3% and 4%. All adult CSLs including yearlings were predominately infected with nematodes, while the pups were mostly infected with acanthocephalans (Table 3).

**Table 3:** Gastrointestinal helminths collected from California sea lions (*Zalophus californianus*) of different age groups from The Marine Mammal Center, California, USA.

Age groups	Trematodes		Cestodes		Nematodes		Acanthocephalans	
	P, %	I, min–max (mean ± SD)	P, %	I, min–max (mean ± SD)	P, %	I, min–max (mean ± SD)	P, %	I, min–max (mean ± SD)
Pups, 8–9 months (n = 10)	–	–	–	–	–	–	10	1
Pups, 10–11 months (n = 12)	25	1–56 (35 ± 30)	33	1–30 (9 ± 14)	67	1–30 (8 ± 10)	100	1–1,226 (155 ± 347)
Yearlings, 1,8 years (n = 4)	25	7	75	1–12 (62 ± 6)	100	1–246 (78 ± 114)	100	19–66 (36 ± 22)
Adults, 3–16 years (n = 8)	63	1–91 (28.6 ± 36)	88	1–43 (20 ± 15)	100	145–1,530 (449 ± 769)	88	3–116 (36 ± 44)

Abbreviations; P – prevalence, I – intensity.

Statistically significant differences in species diversity were not observed between pups and yearlings (Mann-Whitney test: U = 11, z = −1.546; p = 0.122), or between yearlings and adult CSLs (Mann-Whitney test: U = 7, z = −0.626; p = 0.531), while the parasite species diversity in pups and adult seals differed significantly (Mann-Whitney test: U = 9, z = −2.196; p = 0.028).

## 4 Discussion

California sea lions inhabit a large area of the North Pacific with their range encompassing the Pacific coast of North America from British Columbia, Canada to Baja California, Mexico (Carretta et al., 2007). They feed on epipelagic forage fishes in the coastal portions of the California Current Ecosystem and forage on a wide variety of prey. These prey include mainly fishes (Northern anchovy *Engraulis mordax*, Pacific whiting *Merluccius productus*, jack mackerel *Trachurus symmetricus,* rockfish *Sebastes* spp., Pacific mackerel *Scomber japanicus*, blacksmith *Chromis punctipinnis*, opaleye fish *Girella nigricans*, etc.), cephalopods (*Loligo opalescens*, *Onychoteuthis borealijaponicus*, *Abraliopsis* spp., *Gonatus* spp., *Octopus* spp.), and occasionally clams (Lowry et al., 1991). Most of the parasites of CSLs have indirect life cycles that include various species of marine invertebrates and fishes as a source of infection (Marcogliese and Cone, 1997; Marcogliese, 2002; Poulin and Chappell, 2004; Thompson et al., 2005).

In this study we revised the parasite fauna of CSLs based on new material and detailed revision of previously published records (Table 1, Table 2). We concluded that out of the 33 species reported from CSLs, only 24 species could be confirmed as parasites of CSLs. The additional six species represented accidental infections, mainly those of freshwater trematodes which included *Heterophyes heterophyes* (von Siebold, 1853) and two species of *Schistosoma* Weinland, 1858) reported only once from CSLs living in the zoological garden in Giza (Ezzat et al., 1958). Moreover, another trematode, *Stephanoprora denticulata* (Rudolphi, 1802) was found only once in a CSL captured in the National Zoological Gardens, Washington D.C. (Price, 1932). Reports of three additional species are considered to represent misidentifications, namely tapeworm *Adenocephalus* (*Diphyllobothrium*) *pacificus* (in fact a new, undescribed species of *Diphyllobothrium*), heartworm *Acanthocheilonema spirocauda* (Leidy, 1858) identified as microfilaria in blood (Taylor et al., 1961), which is most probably microfilaria of *A. odendhali*, and only a brief record of unidentified nematode of the genus *Dujardinia* Gray, 1858 reported from a CSL living in a zoo (Herman, 1942).

We confirmed the presence of 17 species of metazoan parasites in newly obtained material. Among them 3 species represented new host records and 3 previously unrecognized species (Table 1, Table 2). The nonspecific parasites of CSLs are represented mainly by nematodes that infect various other marine mammals, but also by immature acanthocephalans from the genera *Andracantha* and *Profilicollis* that are parasites of fish-eating birds. These nonspecific acanthocephalan species were found exclusively in young CSLs (pups and yearlings) (Table 1, Table 3). This pattern reflects age-related trends in the development of the parasite fauna of CSLs.

Anisakid nematodes are typical parasites of marine mammals with indirect life cycles using various crustaceans and fishes as a source of infections (McClelland et al., 1990). In the case of maturity, approximately half of *A. simplex s*. *l*. specimens were represented by larvae, while only about a quarter of the specimens of *Pseudoterranova* and *Contracaecum* collected were immature. These data confirm the assumption that CSLs, as well as northern fur seals *Ca*. *ursinus*, are not preferred hosts for *A. simplex*, which is a more typical parasite of cetaceans (Smith and Wootten, 1978; Gaevskaya, 2005). However, the members of the genera *Pseudoterranova* and *Contracaecum* parasitize primarily pinnipeds (McClelland, 2002; Mattiucci and Nascetti, 2008; Kuzmina et al., 2014).

Moreover, finding of *P. azarazi* in this study represented a new host record. This species was previously registered only in Steller sea lions *Eumetopias jubatus* and *Ca. ursinus* in the Northern Pacific (Mattiucci and Nascetti, 2008; Kuzmina et al., 2014). We believe that it is not an accidental infection of CSLs because three CSLs were infected (Table 2).

Three species of the genus *Contracaecum* were previously reported from CSLs (Table 1), including *C. osculatum* that was repeatedly recorded as a pathogen causing gastric ulcerations (Herman, 1942; Sweeney and Gilmartin, 1974; Ridgway et al., 1975; Stroud and Dailey, 1978). Another species, *C. ogmorhini*, was thoroughly re-described from CSLs and other pinniped species (Fagerholm and Gibson, 1987). Later, two sibling species of *C. ogmorhini* were recognized by enzyme electrophoresis and molecular methods (D'Amelio et al., 1994; Zhu et al., 2001). A new species, *C. margolisi,* was described from CSLs based on molecular studies of specimens collected from Vancouver Island, Canada (Mattiucci et al., 2003). However, the authors gave only a brief morphological description, and indicated that it was impossible to distinguish *C. margolisi* and *C. ogmorhini* based on morphological characters. It was suggested that *C. ogmorhini* probably parasitizes pinnipeds in the Southern Hemisphere, while *C. margolisi* – pinnipeds in the Northern Hemisphere (Mattiucci et al., 2003; Shamsi et al., 2009). Morphology of the nematodes found in our study corresponded to that of *C. ogmorhini* from CSLs and other marine mammal species described by Fagerholm and Gibson (1987). Some anisakid nematodes, including *Contracaecum* spp., were shown to be complex species (Mattiucci and Nascetti, 2008), thus we suppose that detailed molecular studies of *C. ogmorhini* collected from CSLs in the present study will give more information concerning taxonomic status of this species.

Besides anisakids, a filariid nematode *Acanthocheilonema odendhali* was found in five CSLs (Table 2). This nematode was also reported from *E. jubatus* and *Ca. ursinus* with slightly higher prevalence (12.5–22.9%) (Dailey and Hill, 1970; Perry and Forrester, 1971; Kagei and Oda, 1975; Kuzmina et al., 2013). The intermediate hosts or vector of this species are unknown, however, according to the results of extensive phylogenetic examination of filariid nematodes by Lefoulon et al. (2015), we suppose that mites or ticks can successfully transmit this species between CSLs. Future ecological studies with application of classical parasitological and new molecular methods are necessary to confirm this assumption.

We did not have an opportunity to examine the lungs of CSLs dissected, therefore, the lung metastrongyloid nematode *Otostrongylus circumlitus* was not found in this study. However, another lung metastrongyloid species *Pa. decorus* was detected in two adult CSLs, but its prevalence and intensity were not determined (Table 2). This species is typically a parasite of young CSLs which eat small opaleye fish (*Girella nigricans*) in shallow waters and intertidal zones; however adult CSLs are also infected (Dailey, 1970).

Tapeworms from several seals have been re-examined in the last decade, but not from CSLs (Hernández-Orts et al., 2015, 2018; Waeschenbach et al., 2017; Kuchta and Scholz, 2017). Previous studies reported only members of the genus *Diphyllobothrium*, mainly *D. pacificum* (now *Adenocephalus pacificus*) without any morphological documentation (Table 1). Our morphological, as well as molecular, studies confirmed that diphyllobothriids from CSLs are not conspecific with *A. pacificus* (Waeschenbach et al., 2017; present study). Additionally, it is the first case (except a brief report by Mariaux et al., 2017) of finding tetrabothriid tapeworms of the genus *Anophryocephalus* in CSLs. Moreover, this is the first record of the species of the genus *Anophryocephalus* out of the Arctic and Subarctic regions (Mariaux et al., 2017). Most of the tapeworms found in this study were immature which corresponds with studies of tapeworms from *Ca. ursinus* (Kuzmina et al., 2015).

Two of three specific trematode species of CSLs were found in this study. *Zalophotrema hepaticum* was found in 4 CSLs, and *Apophallus zalophi* in 6 CSLs (Table 2). Both species were found in all age groups. The third species, the heterophyid *Galactosomum ubelakeri* (Dailey, 1969), was not found. This is probably due to its low prevalence which was previously reported around 7% (Dailey, 1969; Dailey and Hill, 1970).

All other trematode species reported from CSLs represent accidental infections of animals kept in zoos (Price, 1932; Ezzat et al., 1958; see Table 1), except one record of *N. salmincola* (Stroud and Dailey, 1978). This species parasitizes terrestrial mammals, and salmonid fishes serve as its intermediate hosts. Finding this species in CSLs may represent an accidental infection as well as its finding in one *Ca. ursinus* from St. Paul Island, Alaska (Kuzmina et al., 2018).

Ectoparasites are extremely common in seals (Kinne, 1985). The mites of the genus *Orthoharachne* were found in all age categories of CSLs. Usually single mites (mostly nymphs) parasitized pups, whereas dozens and hundreds of mites parasitized adult CSLs. Two distinct species have been found, *O. attenuata* in the nasal cavities of more than 85% of CSLs examined, and *O. diminuata* in trachea and bronchi of about 20%. Both species could be found in the CSLs at the same time. Unfortunately, we did not have an opportunity to examine whole nasal cavities/turbinates and collect all specimens and estimate the intensity of infections. Thus, our data on the prevalence and intensity of the mite infections are roughly estimated. Nevertheless, our data are comparable with the results of Dailey and Hill (1970). These ectoparasites could probably serve as intermediate hosts for the filariid nematode *A. odendhali* (see above).

The mites of the genus *Demodex* Owen, 1843 (Demodicidae) were not found in this present study because visible skin lesions typical for this parasite were not observed; therefore, we did not look for this parasite specifically.

In this study, we had the opportunity to examine the parasite fauna in CSLs of different age groups from 8 months to 16 years. In 2015, in parallel with our studies at TMMC, we examined the parasites of CSL pups of 4–7 months of age (Lyons et al., 2016). Therefore, we had the opportunity to observe the changes in the species composition of the parasite community in CSLs related to age. Hookworms *Uncinaria lyonsi* were found to be the first parasites infecting CSL pups from their first day of life by transmission through their mother's milk (Lyons et al., 2016). At the same time, pups get infected with nasal mites through direct contact with their mothers and other CSLs (Lyons, personal communication). Hookworms parasitize the pups' intestines up to 7–8 months of age, when the pups start independently feeding on fishes and other preys (Lyons et al., 2000, 2016). We did not find *U. lyonsi* in animals at TMMC, because all of these CSLs we studied were older than 8 months.

Our data shown that the acanthocephalans were the first metazoan parasites with an indirect life cycle that infected CSL pups when they switch to independent feeding. In our study, only *C. strumosum* was found to infect the pups which are 8–9 months of age; the prevalence of acanthocephalans increased to 100% in pups 10 months of age (Table 3). We presume that this may happen when pups start feeding independently in shallow waters and eat small crustaceans, which are common intermediate hosts for these acanthocephalans. Other groups of helminths infect young CSLs later, when they begin to swim actively and feed on fishes. The differences in species diversity of the gastrointestinal parasites were found to be statistically significant between pups and adult CSLs. We propose this finding reflects significant differences in the diet of adults that feed on larger fish species as compared to pups.

Despite decades of extensive investigations addressing biology, ecology and diseases of CSLs, their parasites remained superficially studied. The results of this study broaden the knowledge of CSL parasite communities and correct the list of reported parasites to 24, including 3 new host records and 3 new species.

## Compliance with ethical standards

### Conflict interest

The authors declare that there is no conflict of interests regarding the publication of this paper.
